# An Avocado Extract Enriched in Mannoheptulose Prevents the Negative Effects of a High-Fat Diet in Mice

**DOI:** 10.3390/nu14010155

**Published:** 2021-12-29

**Authors:** Paul J. Pistell, Tadanobu Utsuki, Joseph Francis, Philip J. Ebenezer, Jennifer Terrebonne, George S. Roth, Donald K. Ingram

**Affiliations:** 1 Department of Psychology, Towson University, 8000 York Road, Towson, MD 21252, USA; ppistell@towson.edu; 2 Institute for Drug Discovery, Purdue University, 720 Clinic Drive, West Lafayette, IN 47907, USA; tadautsuki@hotmail.com; 3 Comparative Biomedical Sciences, School of Veterinary Medicine, Louisiana State University, Skip Bertman Drive, Baton Rouge, LA 70803, USA; jfrancis@lsu.edu (J.F.); philipje@lsu.edu (P.J.E.); 4 Pennington Biomedical Research Center, Louisiana State University, 5600 Perkins Road, Baton Rouge, LA 70808, USA; jennifer.dowden@pbrc.edu; 5 GeroScience, Inc., 19895 Southern Hills, Baton Rouge, LA 70809, USA; geor@iximd.com

**Keywords:** rare sugar, obesity, glycolysis, metabolism, insulin, adipokines, oxidative stress, mTOR

## Abstract

Beginning at 16 weeks of age and continuing for 44 weeks, male C57BL/6J were fed either a control (CON) diet; a high-fat (HF) diet (60% unsaturated); or the HF diet containing an extract of unripe avocados (AvX) enriched in the 7-carbon sugar mannoheptulose (MH), designed to act as a glycolytic inhibitor (HF + MH). Compared to the CON diet, mice on the HF diet exhibited higher body weights; body fat; blood lipids; and leptin with reduced adiponectin levels, insulin sensitivity, VO2max, and falls from a rotarod. Mice on the HF + MH diet were completely protected against these changes in the absence of significant diet effects on food intake. Compared to the CON diet, oxidative stress was also increased by the HF diet indicated by higher levels of total reactive oxygen species, superoxide, and peroxynitrite measured in liver samples by electron paramagnetic resonance spectroscopy, whereas the HF + MH diet attenuated these changes. Compared to the CON, the HF diet increased signaling in the mechanistic target of the rapamycin (mTOR) pathway, and the addition of the MH-enriched AvX to this diet attenuated these changes. Beyond generating further interest in the health benefits of avocados, these results draw further new attention to the effects of this rare sugar, MH, as a botanical intervention for preventing obesity.

## 1. Introduction

Obesity is viewed as one of the major factors contributing to morbidity and mortality in industrialized nations, and the rate of this condition has been increasing dramatically over the past several years in these populations [1,2]. Many pharmacological interventions to prevent and treat obesity have been developed, with only a few found minimally effective [3]. The following are the current FDA-approved drugs (generic name/brand name) in the U.S.: (a) liraglutide (Saxenda); (b) naltrexone-bupbupropion (Contrave); (c) orlistat (Alli, Xenical); (d) phentermine (Adipex-P, Ionamin, Pro-Fast); and (e) phentermine-topiramate (Qsymia). Additionally, many botanical interventions have been proposed and marketed, but few have strong clinical support [4]. Watanabe et al. [5] provided a recent systematic review of dietary supplements/botanicals claiming to have weight control effects. They rated the quality of the evidence for such claims as low, moderate, or high. None of the candidates were rated to have a high quality of evidence to support claims. Grouped by proposed mechanism of action, the following candidates were rated to have a moderate quality of evidence: (a) Nutrient Absorption: green tea, white kidney beans; (b) Appetite Suppression: whey protein, caffeine; (c) Energy Expenditure: dyacilglycerol; and (d) Carbohydrate Metabolism: chromium.

The current study examined a botanical intervention focusing on a novel approach to obesity prevention, specifically the inhibition of intracellular glucose metabolism. To this end, we utilized an aqueous extract of unripe avocados (AvX) that was enriched in the 7-carbon sugar, D-mannoheptulose (MH) to determine its effectiveness in a mouse model of diet-induced obesity [6]. MH has been discovered in a variety of plants, including alfalfa, fig, and primose [6,7], but is found in high concentrations (1–5% by weight) in unripe avocados [7]. It is a major soluble sugar found in phloem sap, leaf petiole exudates, seed, and mesocarp and is thought to be involved in the ripening process, as levels decrease dramatically with advance ripening [7].

The potential therapeutic benefits of MH were identified in early human studies focused on its potential for improving hypoglycemia [8,9]. Major increases in blood glucose levels could be produced via iv delivery of MH with concomitant decreases in serum insulin levels. Since high levels of MH had been found in unripe avocados, additional experiments were performed to examine the fruit’s effects on the blood/insulin axis [8]. Following consumption of raw avocados, a significant decrease in serum insulin levels w observed without the significant spike in blood glucose. Since MH was assumed to be the active ingredient affecting the insulin levels, it was important to document that the compound could be detected in urine after avocado consumption [8]. MH is available from commercial sources; however, the typical price (~$20/mg) renders use of synthetic forms as prohibitive for long-term animal studies or application to human treatments.

Our interest in MH emerged from the search for effective calorie restriction mimetics (CRM) [10,11,12,13,14]. The objective of CRM is to target the anti-aging and anti-disease effects generally associated with nutritious, low-calorie diets but without an actual reduction in calories consumed. The unique action of MH as a candidate CRM is that this natural compound inhibits glucose metabolism via the glycolytic enzyme, hexokinase, which is the first step in the cellular conversion of glucose to adenosine triphosphate (ATP) and pyruvate [15]. Inhibiting glucose metabolism is proposed to create a cellular response mimicking energy restriction to activate specific genes to increase the efficiency of cellular metabolism and protective mechanisms [14].

Further rationale for and production of the AvX have been described in a recent report [6]. Studies were conducted to demonstrate that following oral consumption of AvX, MH can be detected in both urine and blood in mice and dogs [6]. Moreover, consumption of the AvX reduced circulating levels of glucose and insulin without reductions in food intake [6]. Additionally, in vitro experiments utilizing muscle cells confirmed that the AvX produced molecular signaling events paralleling those reported in in vivo CR studies, such as increased levels of pAMPK, SIRT1, and PGC1α. Given the previous results, we were motivated to investigate the anti-obesity effects of an MH-enriched AvX in a mouse model.

To this end, we investigated whether the effects of long-term feeding (44 weeks) of an HF diet could be attenuated by adding AvX to the diet. We examined effects on body composition, food intake, metabolism, insulin sensitivity, blood lipids, leptin, adiponectin, motor performance, oxidative stress, and mTOR signaling. High-fat diets are well known to induce oxidative stress in many organs [16,17,18]. We utilized electron paramagnetic resonance (EPR) to measure production of reactive oxygen species (ROS). A major biochemical outcome of CR is the effects on the mechanistic target of rapamycin (mTOR) pathway [19,20]. CR inhibits mTOR to produce several downstream effects to activate autophagy, induce catabolism of fats over anabolism of carbohydrates, and decrease oxidative stress and inflammation. In summary, the current results aim to generate additional interest in the health benefits of avocados but also present a consideration of a novel mechanism, i.e., glycolytic inhibition, that can be applied as a botanical intervention for prevention of obesity.

## 2. Methods

### 2.1. Avocado Extract

The method for producing an aqueous extract of unripe avocados has been reported previously [6]. Operating under the direction of the Iams Company (later P&G Pet Care, Dayton, OH, USA), the general procedure followed by the manufacturer, Kemin Industries (Omaha, NE, USA), can be described briefly. Processed in batches of 900 kg, whole avocado fruit of the Hass variety was obtained from a California grower in an unripened state and frozen until the manufacturing of the AvX was initiated. The whole fruit (flesh, peel, and pit) was placed into a disintegrator, followed by agitation and then the addition of water (about 3000 kg), along with ~1 L of a cellulase preparation (Cellubrix, Novozymes, Denmark). Continuous agitation was applied to the mixture and was concurrently heated to about 66 °C. Upon reaching this temperature, additional Cellubrix (~1 L) was added. The mixture remained under agitation for about 12 h at a controlled pH of about 5.5. The temperature was then increased to about 80 °C and held at this level for about 2 h whereupon the resulting digested plant mixture was then filtered to provide the carbohydrate extract as the filtrate. The carbohydrate extract was then evaporated in a simplified recirculation system at 80 °C, under vacuum, to provide the carbohydrate extract having around 10–20% solids with a pH of about 5.5. Using a refractance window dryer, the extract was then further concentrated to provide about 100 kg of the extract as a powder with a yield of about 11% carbohydrate extract, based on the starting mass of the whole avocado fruit. As estimated by liquid chromatography–mass spectrometry, the yield of MH in the AvX used in the experiments presented in the current report was estimated to be about 14%., which compares to an estimate of ~1% MH normally found in the Hass cultivar [21]. Other macronutrient amounts are presented in Table 1. After being manufactured, the AvX was stored at −20 °C prior to its application to the diet used.

### 2.2. Animals and Diets

Male C57BL/6J mice around 90 days of age were purchased from the Jackson Laboratory (Bar Harbor, ME, USA) and housed four per cage in the barrier facility at the Pennington Biomedical Research Center (PBRC, Baton Rouge, LA, USA) in ventilated racks (Lab Products, Seaford, DE, USA). Bedding consisted of sterilized corn cob bedding, and bedding and cages were changed under a ventilated hood every week. Sterilized and acidified water was provided in sterilized bottles that were changed weekly. The rooms were environmentally controlled to provide a 12-h:12-h dark cycle (with lights off at 18:00), 32 ± 2 °C, and 55 ± 2% relative humidity. The mice were provided the AIN-93G diet (Catalog #: 7597; Dyets, Bethlehem, PA, USA) until 12 weeks of age and were then randomly assigned by cages to one of three diet groups (*n* = 16) as follows: (1) Control (CON) maintained on the AIN-93G diet; (2) High-Fat (HF) fed a customized version of the AIN-93G diet which contained hydrogenated coconut oil to provide 60% of calories from fat (Catalog #101920; Dyets, Betheleham, PA, USA); High-Fat with the MH-enriched avocado extract (HF + MH), which was the same as the HF group but with the AvX added to provide an estimated daily dose of 1.7 g/kg MH to each mouse. This dose was selected because it was highly effective in preventing growth of tumor cells in a rat model [22]. All diets were provided ad libitum (AL) and continued for 44 weeks. Half of the mice were sacrificed at 5.5 months on the diets, while the remaining mice were sacrificed at the end of the treatment period, and various tissue samples were obtained and stored at −70 °C until time of assay. 

The animal protocol (#538) applied was reviewed and approved (8 May 2008) by Institutional Animal Care and Use Committee of the Pennington Biomedical Research Center according to the U.S. Public Health Service’s Guide for the Care and Use of Animals.

### 2.3. Body Composition and Food Intake

Mice were assessed for body composition using NMR (Brucker Minispec MQ10, Bilerica, MA, USA). The mice were inserted unanesthetized into a plastic cylinder which was placed inside a bore for approximately 2–3 min. This assessment was done at baseline, and 2.5, 5, and 9 months after the start of the treatment. To measure food intake, food was placed into the food hopper of the cage at the beginning and the end of each week. Estimates were made of individual food intake by dividing the totals by the number of mice in the cage. This method of monitoring food intake by cage did not account for spillage/wastage of the food. For a more accurate estimate of individual food intake, mice were removed from their group cages and housed individually in a hanging cage permitting food waste to be collected from the tray below. Beginning at 3 weeks into experiment, mice were housed in this situation for 2 weeks and then returned to their group housing.

### 2.4. Metabolism

Several measures of metabolism were obtained by indirect calorimetry using the CLAMS system (Columbus Instruments, Columbus, OH, USA) which also contained a set of infrared beam detectors allowing monitoring of locomotor activity. Beginning at 40 weeks into the experiment, individual mice were given 3 days of habituation to the chambers in the apparatus, after which the chambers were cleaned. Various measures were then obtained over the next 4 days without disturbing the mice.

### 2.5. Insulin Sensitivity

After 3 months on the diet, a measure of blood glucose was obtained by nicking the tail of each mouse with a sterile, sharp razor blade and collecting the blood sample to be read by a glucometer (Ascensia Elite, Bayer, Mishawaka, IN, USA). Following this blood sample to obtain a baseline measure of glucose, each mouse was then injected IP with a dose of 0.75 U/kg insulin from a working solution of 0.1 U/mL. Blood samples were withdrawn at 0, 15, 30, 60 and 90 min using the tail nick protocol, and the samples were measured for glucose using the glucometer. These procedures were repeated after the mice had been on their respective diets for 9 months.

### 2.6. Blood Lipids

A blood sample was obtained during tissue collection after 22 and 44 weeks on treatment to measure blood lipids. Whole blood was collected using cardiac puncture while mice were anesthetized prior to euthanasia. Measures of total cholesterol and triglycerides were made using the CardioChek system (PTS Diagnostics, Whitestown, IN, USA).

### 2.7. Oxidative Stress

Using electron paramagnetic resonance (EPR) spectroscopy as previously described [23,24], various measures of oxidative stress were collected from liver samples obtained during necropsy conducted after the mice were euthanized. Briefly, two different spin probes were used for EPR studies. The compound 1-hydroxy-3-methoxycarbonyl-2, 2, 5, 5-tetramethylpyrrolidine (CMH) was used to measure total reactive oxygen species (ROS) and total superoxide (O2^•−^) levels, while 1-hydroxy-3-carboxypyrrolidine (CPH) was used to measure peroxynitrite (OONO^−^) levels. All EPR measurements were performed using an EMX EPR eScan BenchTop spectrometer and a super-high-quality factor (Q) microwave cavity (Bruker Company, Bellerica, MA, USA).

For measurement of ROS, tissue samples from each mouse were minced and placed into four wells of a 24-well plate with 20mM KHB containing 25 µM DF and 5 µM DETC. Tissue pieces were then washed twice with the same buffer to remove any trace contamination, and were incubated at 37 °C with specific spin probes for 30 min. The incubation of tissue was terminated by placing the plate on ice. Detection of ROS and oxygen concentration was conducted under the following EPR settings: center field g = 2.002; field sweep 50 G; microwave power 20 mW; modulation amplitude 1.90 G; conversion time 10.24 ms; and time constant 81.92 ms. For measuring O2^•−^, tissue pieces were incubated at 37 °C with PEG-SOD (50 U/mL) for 30 min, and then the spin probe CMH (200 μM) was added for another 30 min incubation period. Aliquots of the incubated probe media were then added to 50 µL glass capillary tubes for determination of total superoxide production. Preincubation of tissue with PEG-SOD allows competitive inhibition of CMH by intracellular and extracellular released O2^•−^. Since it is cell permeable, PEG-SOD can competitively inhibit the CMH/O2^•−^ interaction in both the intracellular and extracellular spaces, thus allowing accurate measurement of tissue O2^•−^ production. For tissue O2^•−^ production, the values obtained from incubation with PEG-SOD and CMH were subtracted from the values obtained from incubation with CMH only. For determination of O2^•−^ production, the above mentioned EPR settings were used. For OONO^−^ production, tissue pieces were incubated at 37 °C with 30 µL of 500 μM CPH for 30 min. Aliquots of the incubated probe media were placed into 50-µL disposable glass capillary tubes (Noxygen Science Transfer and Diagnostics) for determination of OONO^−^ production.

### 2.8. Western Blot for mTOR Signaling and ELISA for Leptin and Adiponectin

The adipose tissues collected following euthanasia were homogenized in a lysis buffer (4 mM MOPS, 0.4 mM EGTA, 1 mM EDTA 0.5% *v*/*v* Nonidet P-40) containing various protease (phenylmethylsulphonylfluoride, leupeptin pepstatin A, and aprotinin) and phosphatase (sodium vanadate, sodium fluoride, sodium pyrophosphate, β-glycophosphate, and benzamifin) inhibitors. The aqueous and lipid phases were separated by centrifugation with 12,000× *g* three times. The aqueous phase was subjected for determinations of mTOR signaling by western blot and leptin/adiponectin by sandwich ELISA. For western blot, extracted materials were heated in a sample buffer (65 mM Tris-HCl, 2%*w*/*v* SDS, 10% *v*/*v* glycerol, 2% *v*/*v* β–mercaptoethanol). Proteins (5–20 μg) were resolved by SDS-PAGE using Tris-Glycine gel (Invitrogen, Carlsbad, CA, USA) and transferred to PVDF. All primary antibodies were purchased from Cell Signaling (Temecula, CA, USA). All membranes were immunoblotted by following the manufacturer’s instructions. The signals were detected by chemiluminescence (GE Healthcare Bio-Sciences Corp, Piscataway, NJ, USA) and exposed to film. Levels of leptin and adiponectin were determined using sandwich ELISA (R&D Systems, Minneapolis, MN, USA), following the manufacturer’s instructions.

### 2.9. Statistical Analysis

Data were analyzed using ANOVA, followed by Tukey post hoc tests when appropriate to compare the HF and HF + MH groups to the CON group. The data were analyzed using Statistical Package for the Social Sciences (SPSS), and 0.05 was set as the significance level.

## 3. Results

### 3.1. Body Composition and Food Intake

**Figure 1:**
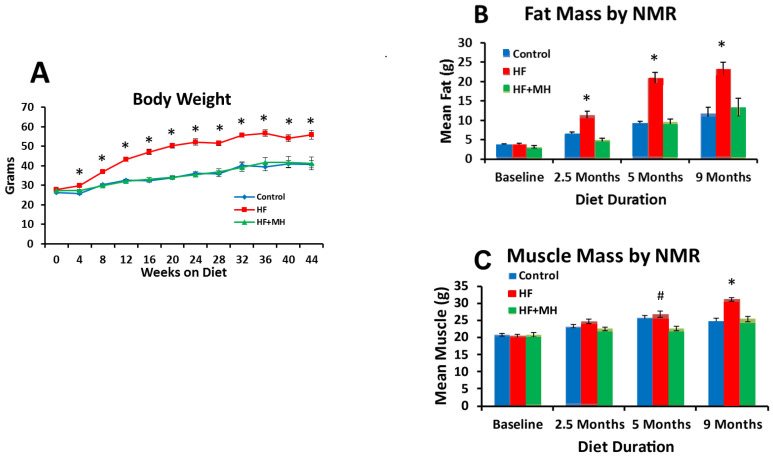
(**A**): Comparison of mean body weights by month for mice across the duration of the study (Ns = 7–8). (**B**): Comparison of fat mass by NMR at baseline and after 2.5 and 5 months on diets (Ns = 12–16), and 9 months on diets (Ns = 7–8). (**C**): Comparison of muscle mass by NMR at baseline and after 2.5 and 5 months on diets (Ns = 12–16), and after 9 months on diets (Ns = 7–8). # = *p* < 0.05 & * = *p* < 0.01 compared to controls. HF: high-fat, MH: mannoheptulose.

As expected, the HF diet induced significant gains in body weight from baseline, showing statistical significance beginning at 4 weeks on the diet compared to the CON group (Figure 1A). In fact, body weight in these mice nearly doubled from baseline, whereas the increase in weight in the CON group was less than 30% compared to baseline. Most interestingly, the trajectory of body weight gain in the HF + MH group mirrored that of the CON group throughout the study period.

As measured by NMR, fat composition was also significantly impacted by diet (Figure 1B). In mice on the HF diet, fat content increased significantly compared to the CON, beginning at 2.5 months on diet and continuing through 9 months. In contrast, the HF + MH group did not differ significantly in fat mass from the CON group at any time-point. Figure 1C shows an increase in muscle mass in the HF group compared to the CON, significantly different at 9 months. No significant differences in muscle mass were observed between the HF + MH group and CON.

**Figure 2:**
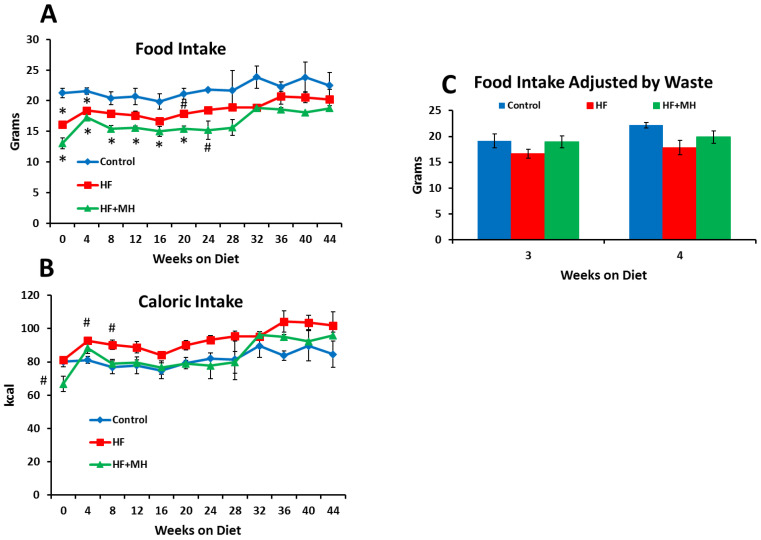
(**A**): Weekly food intake per mouse at weekly intervals on the diets. (Ns = 7–8). (**B**): Weekly caloric intake at weekly intervals on the diets. (Ns = 7–8). (**C**): Weekly food intake per mouse at weeks 3 and 4 on the diets adjusted for food waste, with mice housed individually in suspended cages (Ns = 8–10). # = *p* < 0.05 & * = *p* < 0.01 compared to controls.

Figure 2 presents data on food intake from a couple of perspectives. First, in Figure 2A, it can be noted that CON mice actually consumed significantly more of their food measured by weight compared to the HF group and the HF + MH group during the early part of the study. There are a couple of explanations for this observation. First, there was less food wastage observed in the cages of the HF groups compared to the CON. Second, the HF diets were much more calorically dense compared to the CON. To this point, results shown in Figure 2B reveal that caloric intake in the HF group was significantly higher compared to the CON group during the first couple of weeks of the study. Moreover, after an initial period of adjustment, the caloric intake of the HF + MH group increased and did not differ significantly from the CON or the HF group for the remainder of the study period. Notably, over the course of last few weeks of the study, no significant group differences were observed in grams of food or calories consumed.

A more accurate estimate of food intake was obtained during weeks 3–4, when the mice were housed individually in hanging, suspended cages, and food waste could be collected and measured. Figure 2C shows that no significant differences in food intake were observed among the groups when measured under these conditions. Thus, we can conclude that the differences in body composition observed between the HF and the HF + MH groups were not due to differences in food intake.

### 3.2. Metabolism

**Figure 3:**
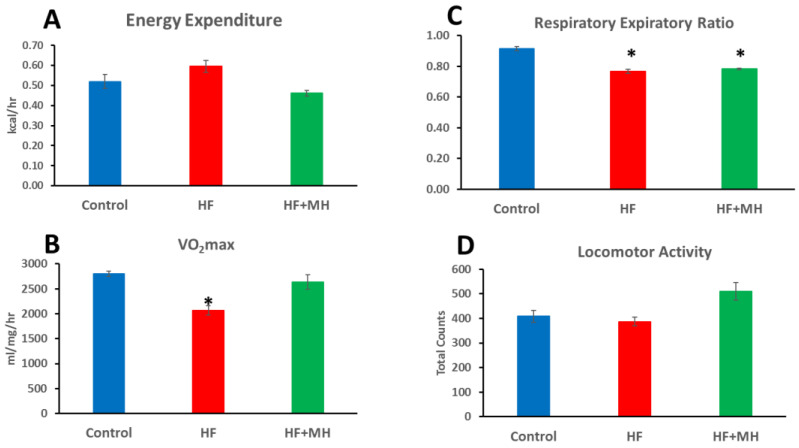
(**A**): Energy expenditure (kcal/hour) over 4 days in CLAMS. (**B**): VO_2_max (oxygen usage) over 4 days in CLAMS. (**C**): Respiratory expiratory ratio over 4 days in CLAMS. (**D**): locomotor activity over 4 days in CLAMS. * = *p* < 0.01 compared to Control group. (Ns = 4.).

Beginning 40 weeks after starting treatment, mice were evaluated for metabolic measures in the CLAMS system for 4 days, as summarized in Figure 3. Mice on the HF diet had significantly lower VO_2_max (B) and respiratory exchange ratio (C) compared to the CON group. The former result indicates the reduced rate of oxygen consumption in mice in the HF group, while the latter result indicates the higher level of fat metabolism in these mice. Mice on the HF + MH diet did not differ significantly from the CON group except having a reduced respiratory ratio (C). Locomotor activity appeared higher in HF + MH group, but the difference did not prove statistically significant.

### 3.3. Glucose Levels and Insulin Sensitivity

**Figure 4:**
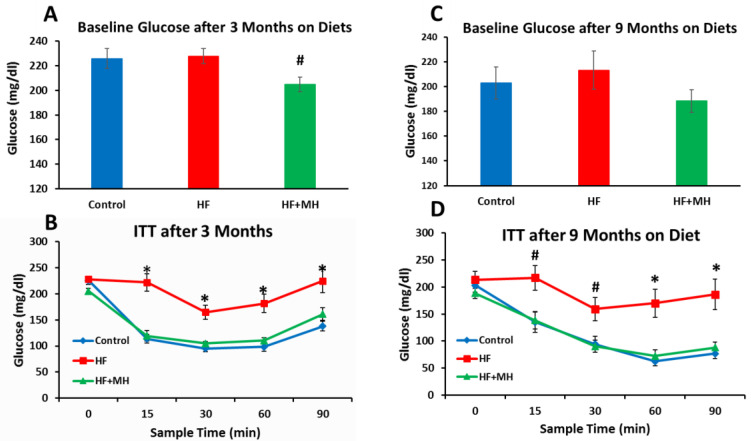
(**A**): Baseline glucose after 3 months on diets (Ns = 15–16). (**B**): Insulin tolerance test (ITT) after 3 months on diets (Ns = 15–16). (**C**): Baseline glucose after 9 months on diets (Ns = 7–8). (**D**): ITT after 9 months on diets (Ns = 7–8). * = *p* < 0.01 compared to controls. # = *p* < 0.05 compared to controls.

All glucose and insulin sensitivity testing was conducted in the same mice throughout the duration of the study. Surprisingly, the blood glucose levels of the mice on the HF diet did not differ significantly when measured at 3 and 9 months after treatment (Figure 4A,C). However, the blood glucose levels of the HF + MH group were significantly lower compared to the CON group at the 3-month time-point (Figure 4A). As expected, the HF diet significantly impaired insulin sensitivity compared to mice on the CON diet at both 3 and 9 months after the start of treatment (Figure 4B,D). In contrast, mice on the HF + MH diet exhibited glucose responses identical to the CON mice after injection with insulin at both time-points of treatment.

### 3.4. Blood Lipids

**Figure 5:**
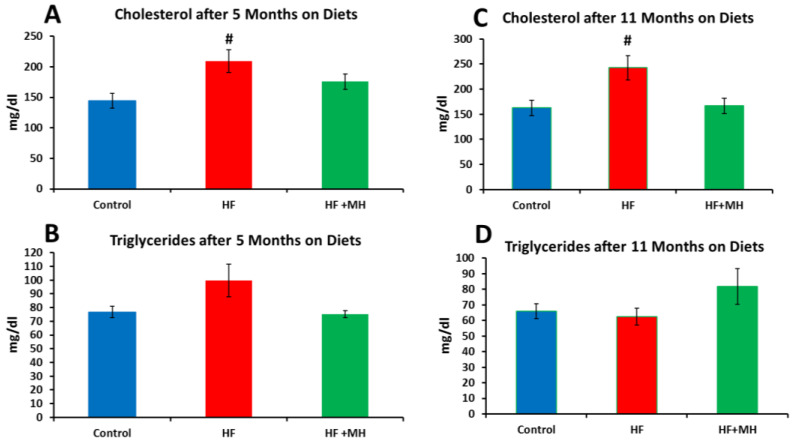
(**A**): Cholesterol measured after 5 months on diets. (**B**): Triglycerides measured after 5 months on diets. (**C**): Cholesterol measured after 11 months on diets. (**D**): Triglycerides measured after 11 months on diets. At 5 months Ns = 5–8. At 11 months Ns = 7–8. # = *p* < 0.05 compared to controls.

Mice on the HF diet had significantly higher cholesterol but not triglyceride levels compared to mice in the CON group (Figure 5). The lipid levels of mice on the HF + MH diet did not differ significantly from those of the CON group.

### 3.5. Leptin and Adiponectin

**Figure 6:**
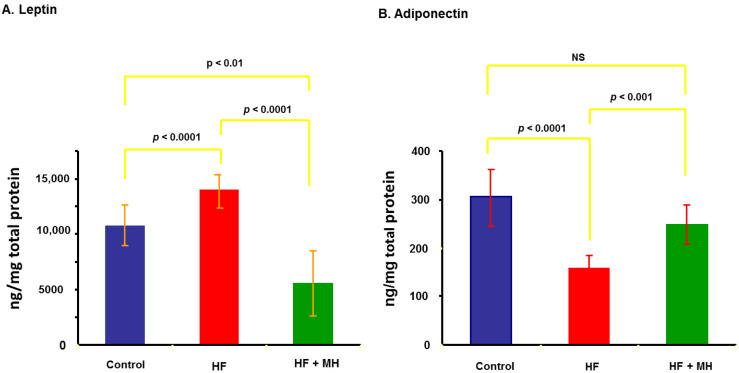
(**A**): Leptin levels measured in white adipose tissue after 9 months on diets (Ns = 6–8). (**B**): Adiponectin levels in white adipose tissue after 9 months on diets (Ns = 6–8).

Leptin is an adipokine that is typically upregulated in animals on high-fat diets. As presented in Figure 6A, the HF diet significantly increased leptin in white adipose tissue compared to the CON diet. Leptin levels in mice on the HF + MH diet were significantly lower compared to both the HF and CON diets. In contrast, the HF diet significantly reduced adiponectin levels compared to the CON (Figure 6B). Mice on the HF + MH diet had significantly higher levels of adiponectin compared to the HF diet but did not differ from the CON mice.

### 3.6. Rotarod

**Figure 7:**
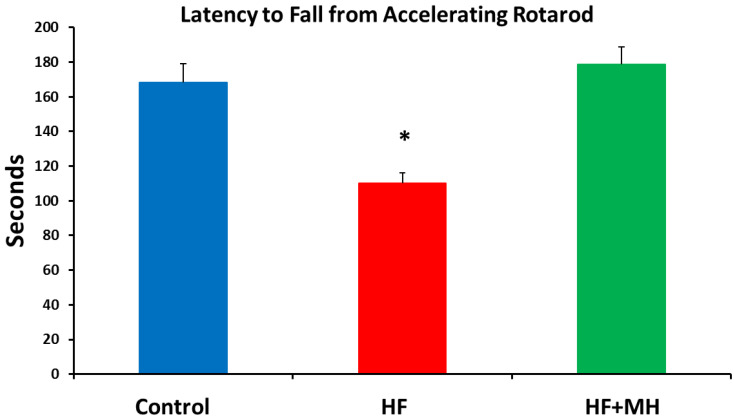
Comparison of diets effects on latency to fall from accelerating rotarod. (Ns = 15–16). * = *p* < 0.01 compared to controls.

The addition of AvX to the HF diet led to functional improvements. As observed in Figure 7, performance in the rotarod task was impaired in the mice on the HF diet compared to the CON and HF + MH groups.

### 3.7. Oxidative Stress

**Figure 8:**
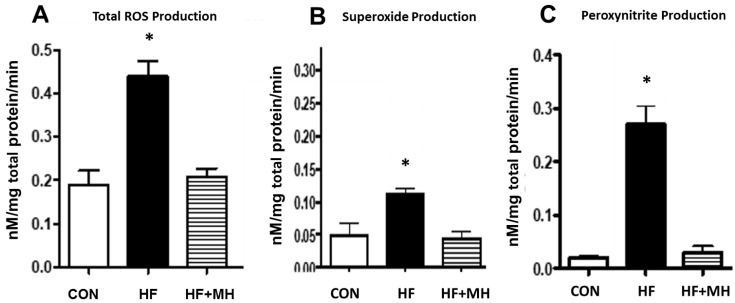
(**A**): Total reactive oxygen species (ROS) production; (**B**): superoxide production; (**C**): peroynitrite production measured by EPR in liver tissue of mice on diets for 9 months; Ns = 6–8; * = *p* < 0.001.

Chronic consumption of a high-fat diet can induce oxidative stress in many organ systems [16,17,18]. As shown in Figure 8, the HF diet induced major increases in three measures of oxidative stress in the liver as measured by EPR. Compared to the CON group, mice on the HF diet exhibited significantly higher levels of total ROS (A), superoxide (B), and peroxynitrite (C). The HF + MH diet totally attenuated the increase in all three measures observed in mice on the HF diet, comparable to levels in the CON group.

### 3.8. mTOR Pathway

**Figure 9:**
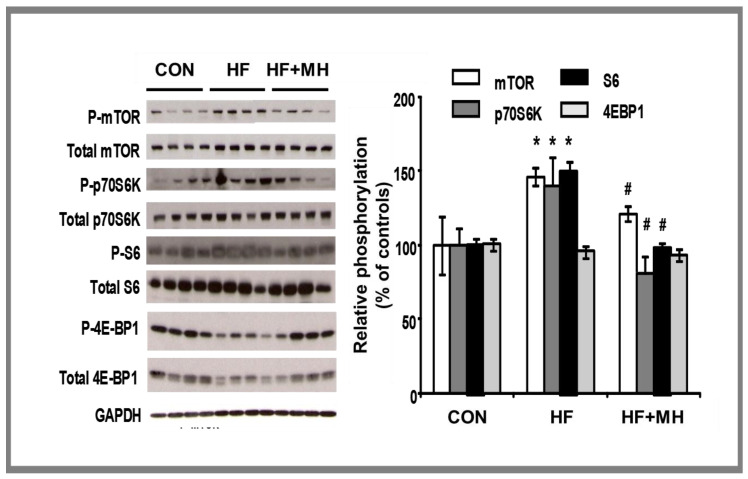
Protein levels of signaling events in the mTOR pathway (S6, p70S6K, 4EBP1) measured in white adipose tissue in mice after 9 months on diets. # = *p* < 0.05, *= *p* < 0.01, compared to controls (Ns = 4).

We examined diet effects on signaling of mTOR in white adipose tissue along with other downstream events in this molecular pathway. Inhibition of mTOR results in downstream phosphorylation of other signaling events, specifically, S6K1, P70S6K, and 4EBP1. As presented in Figure 9, mice on the HF diet exhibited significantly higher yields of these proteins compared to the CON, except for 4EBP1. Mice on the HF-MH diet had significantly lower yields compared to the HF group. This resulting molecular pattern would be indicative of improvements in cellular autophagy.

## 4. Discussion

Given the increasing numbers of persons in the Western world classified as overweight or obese, a great need exists for effective anti-obesity interventions. Several pharmaceutical and botanical products are available on the market, but efficacy remains a major issue [3,4,5].

Several past studies have used mouse models of diet-induced obesity to report similar anti-obesity effects of other fruits or their derivatives. Among examples are the following: lingonberry [25], chokeberry [26], bitter melon [27], mango [28], jamun [29], and kumquat [30]. None of these candidates have been advanced to clinical testing. When treated as the whole fruit, ripened avocado has proven in clinical trials to have several beneficial health effects for overweight or obese individuals. Park et al. [31] reported that the addition of avocado to a breakfast meal lowered concentrations of triglyceride-rich lipoproteins and increased concentration of high-density lipoprotein particles and improved endothelial function. Providing a daily avocado to overweight and obese individuals for 5 weeks, Wang et al. [32] observed reductions in several parameters related to low density lipoproteins. In a related finding, Wang et al. [33] reported reduced levels of oxidized lipoproteins in the same group provided a similar regimen of avocado consumption. Edwards et al. [34] noted improved cognitive function in overweight and obese individuals receiving meals supplemented with avocado for 12 weeks compared to those receiving an isocaloric control diet. Interestingly, none of these clinical studies reported any significant changes in body weight or body composition resulting from avocado consumption.

The current report provides preclinical evidence of the efficacy of an extract of unripe avocados enriched in the rare sugar MH to attenuate the pathophysiology associated with an unhealthy HF diet in mice. As expected over the course of 12 weeks of treatment, the HF diet compared to the CON diet induced higher levels of the following parameters: body weight, body fat, blood lipids, leptin, markers of oxidative stress, signaling in the mTOR pathway, and time spent on the rotarod. Lower levels of the following parameters were observed in the HF group compared to the CON group: insulin sensitivity, adiponectin, VO_2_max, and RER. The addition of the MH-enriched AvX to the HF diet significantly blocked all of its negative health consequences without affecting food intake. Thus, this report adds to the literature describing various botanicals contained within avocados that offer important health benefits.

A recent investigation has used the mouse model of diet-induced obesity to identify a novel product from avocados, avocatin [35], which is a mixture of polyhydroxylated fatty alcohols. Mice were placed on an HF diet for 5 weeks that produced the expected pattern of pathophysiology compared to mice on a nonfat control diet. Those mice on the HF diet that were given oral treatment with avocation B twice weekly exhibited improved glucose tolerance, glucose utilization and insulin sensitivity, as well as modest reductions in body weight and body fat by the end of the study. Based on these findings from the mouse study in addition to a positive safety profile achieved in a small clinical trial described in the same report, avocatin B is now marketed as a daily supplement (Avolean; www.Sentalife.com (accessed on 14 December 2021)). The product is advertised to “help maintain a healthy weight by boosting metabolism,” although no effects on body weight or composition were reported from the clinical study.

The adverse effects of the HF diet observed in the mouse study of avocatin B were more dramatically attenuated in the current study providing AvX to mice on an HF diet. The targeted mechanisms are different. Avocatin B is purported to be a potent inhibitor of fatty acid oxidation [35], as supported by in vitro experiments, whereas the AvX has been shown to increase fatty acid oxidation in vitro [6]. The intended target of the MH in AvX is hexokinase inhibition [6]. This feature makes MH delivered in the AvX a unique botanical candidate for obesity prevention.

Our interest in glycolytic inhibition emerged from a search for effective CRM. Reducing flux through the intracellular glycolytic pathway was hypothesized to induce many of the molecular and cellular responses produced by long-term CR. Loss of body weight is one of the expected outcomes of CR, but we had not proposed that an effective CRM would necessarily result in reduced body weight [36]. It is important to note that the current results demonstrate prevention of diet-induced increase in body weight rather than reduction in body weight.

Other candidate CRMs have been proposed that act on mechanisms of glucose metabolism. Acarbose is a well-developed treatment for type 2 diabetes that, when fed chronically to mice, can increase lifespan at the doses applied [37,38]. The presumed mechanism is inhibition of alpha-glucosidase in the gut which reduces availability of glucose for absorption. Treatment with this compound does reduce body weight in mice and also decreases circulating levels of insulin and IGF-1; however, blood levels of glucose are actually increased, as is food intake, compared to controls. A follow-up study of acarbose showed that glucose tolerance was increased [39].

Although widely used as a nutritional supplement to treat arthritis, glucosamine is another candidate CRM acting as glycolytic inhibitor. In its phosphorylated form, glucosamine is a hexokinase inhibitor with high activity, directed toward glucokinase or HK-1, the isoform with a heavy concentration in the liver. First proposing glucosamine as CRM, Weimer et al. [40] reported increased lifespan in nematodes and mice treated with glucosamine. Glucosamine treatment has also been applied in a couple of studies using rodent obesity models. Studies in rats [41] and mice [42] delivered glucosamine via their drinking water while being fed an obesogenic diet. Over 20 weeks of treatment, improvements in glucose responses were observed to control fed animals without significant effects on body weight. Hawang et al. [42] noted differential effects of glucosamine treatment on body weight in mice depending on the diet. They noted reduced body weight gain in mice provided glucosamine on an HF diet accompanied by improved glucose/insulin responses compared to mice on the same diet without glucosamine. However, in mice fed a control diet, glucosamine treatment resulted in increased body weight accompanied by impaired insulin responses. Our current results applying MH-induced glycolytic inhibition produced clear anti-obesity effects.

Some previous studies applying a similar formulation of MH-enriched AvX in studies of dogs and cats have produced results that are incongruent with those of our mouse study. Ranging from 14 to 42 days of treatment delivering MH doses from 2 to 8 mg/kg, investigations of Labrador Retrievers, Beagles, and domestic cats found no significant effects on circulating levels of glucose or insulin nor on body weight [43,44,45,46,47]. Several significant physiological effects of feeding AvX were observed, however. For example, increased energy expenditure was observed in cats provided AVX at a MH dose of 8 mg/kg for 28 days [43] and in Beagles fed AvX at the same dose of MH for 14 days [44]. In a later study in Beagles, McKnight et al. [45] observed significantly increased levels of fasting serum glucagon-like peptide-1 and post-prandial serum ghrelin, which, long term, should affect the dogs’ appetite. In Labrador Retrievers fed an AvX diet delivering 2 mg/kg of MH for 22 days, McKnight et al. [46] observed significantly increased fasting RQ and glucose oxidation measured by calorimetry. In another study of Labrador Retrievers eating an AvX diet delivering 6 mg/kg of MH for 9 weeks, McKnight et al. [47] also observed significantly reduced post-prandial RQ. It is uncertain why differences between these past studies in dogs and cats and the current findings in mice occurred regarding the effects of AvX on glucose and insulin levels. Among possible explanations are the following: (1) the current mouse study employed much higher concentrations of MH in AvX, (2) current results were based on comparisons to a high-fat diet, (3) the treatment period for mice was much longer compared to the dog and cat studies, and (4) a number of other design issues may have complicated interpretation as discussed previously [6].

## 5. Conclusions

In sum, we report that an MH-enriched extract of unripe avocados offers impressive anti-obesity effects in a mouse model. The study offers new potential for botanical treatment of obesity. Additional work is planned to evaluate the most effective doses of MH and to demonstrate that MH is the active ingredient in AvX producing the favorable effects on the pathophysiology produced by the HF diet.

## Figures and Tables

**Table 1:** Macronutrient Content of Avocado Extract (AvX).

Macronutrient	% (*w*/*w*)
Protein	3.3
Moisture	4.4
Ash	10.2
Fat	10.7
Sugars	
Mannoheptulose	13.7
Perseitol	8.1
Glucose	0.5
Fructose	0.9

## Data Availability

Data supporting reported results can be obtained by emailing Donald K. Ingram at donald.ingram@pbrc.edu.
